# Impact of interfacial molecular orientation on radiative recombination and charge generation efficiency

**DOI:** 10.1038/s41467-017-00107-4

**Published:** 2017-07-19

**Authors:** Niva A. Ran, Steffen Roland, John A. Love, Victoria Savikhin, Christopher J. Takacs, Yao-Tsung Fu, Hong Li, Veaceslav Coropceanu, Xiaofeng Liu, Jean-Luc Brédas, Guillermo C. Bazan, Michael F. Toney, Dieter Neher, Thuc-Quyen Nguyen

**Affiliations:** 1 Center for Polymers and Organic Solids, University of California, Santa Barbara, CA 93106 USA; 2 Institute of Physics and Astronomy, University of Potsdam, Potsdam, 14476 Germany; 3 Materials Department, University of California, Santa Barbara, CA 93106 USA; 4 Stanford Synchrotron Radiation Lightsource, SLAC National Accelerator Laboratory, Menlo Park, CA 94025 USA; 5 Department of Electrical Engineering, Stanford University, Stanford, CA 94305 USA; 6 School of Chemistry and Biochemistry and Center for Organic Photonics and Electronics, Georgia Institute of Technology, Atlanta, GA 30332 USA

## Abstract

A long standing question in organic electronics concerns the effects of molecular orientation at donor/acceptor heterojunctions. Given a well-controlled donor/acceptor bilayer system, we uncover the genuine effects of molecular orientation on charge generation and recombination. These effects are studied through the point of view of photovoltaics—however, the results have important implications on the operation of all optoelectronic devices with donor/acceptor interfaces, such as light emitting diodes and photodetectors. Our findings can be summarized by two points. First, devices with donor molecules face-on to the acceptor interface have a higher charge transfer state energy and less non-radiative recombination, resulting in larger open-circuit voltages and higher radiative efficiencies. Second, devices with donor molecules edge-on to the acceptor interface are more efficient at charge generation, attributed to smaller electronic coupling between the charge transfer states and the ground state, and lower activation energy for charge generation.

## Abstract (web summary)

Molecular orientation profoundly affects the performance of donor-acceptor heterojunctions, whilst it has remained challenging to investigate the detail. Using a controllable interface, Ran et al. show that the edge-on geometries improve charge generation at the cost of non-radiative recombination loss.

## Introduction

The efficiencies of charge generation and recombination at a donor acceptor heterojunction depend on parameters, such as distance and molecular orientation of the donor and acceptor molecules at the interface. These processes dictate the performance of electronic devices such as light emitting diodes (LEDs), photodetectors, and photovoltaics. It is therefore critical to understand the properties of the donor/acceptor interface which affect the efficiencies of charge generation and recombination. The properties of the donor/acceptor interface can be studied from the point of view of photovoltaics, with implications on the performance of other devices which depend on donor/acceptor interfaces.

A fundamental issue under much debate in the organic photovoltaic (OPV) literature involves the geometry of the donor/acceptor interface: whether a face-on geometry (one where the *π*-faces of the donor and acceptor *π*-conjugated molecules or polymer chains are parallel) is favorable compared to an edge-on geometry (where the *π*-faces are orthogonal). Theoretical calculations have long suggested that the nature of the donor–acceptor interface will have a large effect on the rates of charge transfer and recombination^1–3^, as well as charge delocalization^3, 4^. Other calculations have found that molecular orientation affects interfacial quadrupoles and consequently the ease of charge separation^5, 6^. A number of researchers have attempted to resolve this question experimentally with the use of controlled donor and/or acceptor orientations in planar heterojunction solar cells^1, 7–14^. In general, most studies have found that within the same material system, face-on solar cells have a superior power conversion efficiency (PCE) when compared to the edge-on orientation^7, 15^. This has been attributed primarily to changes in the donor ionization potential (IP) (or, to a first approximation, highest occupied molecular orbital energy level), which directly affects the open circuit voltage (*V*_OC_)^7–9, 11, 15–17^, but has also been explained by differences in recombination rates^1, 7, 12, 15^.

Despite significant efforts, it has remained very challenging to fabricate high-quality planar heterojunctions with identical active layers and contacts, but opposite molecular orientations. Most studies settle for comparisons between one orientation and a mixed orientation, or modified contacts to induce changes in orientation. Furthermore, it was demonstrated that molecular diffusion in planar heterojunctions can happen spontaneously^18^, and to varying extents for different orientations^19^. Interfacial mixing will lead to unfair comparisons and erroneous conclusions if not properly taken into account. Furthermore, differences due to interfacial and/or bulk disorder have also been shown to influence device performance^20, 21^. To date, experimental studies in which the effects of molecular orientation have truly been isolated—a single materials system in which the two extremes of face-on and edge-on orientations can be accessed while maintaining abrupt donor/acceptor interfaces and identical contacts—have not been reported.

From the point of view of molecular orientation, our study addresses two topics that have been gaining significant attention in the OPV literature: non-radiative recombination losses to the *V*_OC_^3, 22–27^, and the driving force for charge generation^28–30^. It is thought that non-radiative recombination plays a significant role in efficiency losses in photovoltaics and LEDs, and it is only when non-radiative pathways have been eliminated that organic solar cells become competitive with high performance inorganic materials^23, 25, 31, 32^. A recent theoretical study by Chen et al. on pentacene/C_60_ interfaces predicts that face-on interactions result in less non-radiative recombination, due to reduced vibronic coupling between the charge transfer (CT) state and the ground state (GS)^3^. To date, little is understood about the origin of non-radiative recombination in organic solar cells, how it relates to molecular orientation, or how to curtail this recombination pathway. Furthermore, the driving force for charge generation has remained a disputed topic in the literature, with researchers quoting the need for energetic offsets^33, 34^, hot charges^35, 36^, delocalization^37, 38^, low reorganization energies^39^, electric fields^40, 41^, energetic cascades and disorder^42, 43^, and entropy^29, 44^, to achieve efficient charge generation.

**Fig. 1:**
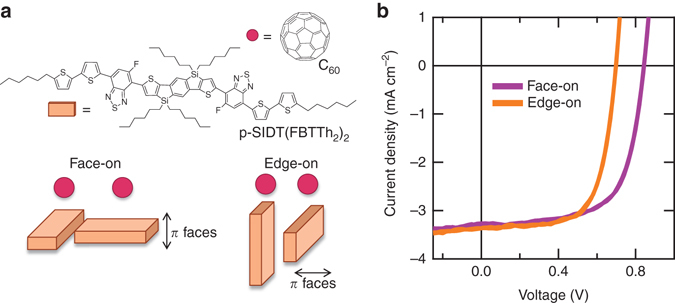
Molecular orientation and the resulting photovoltaic performance. **a** Molecular structures of p-SIDT(FBTTh_2_)_2_ and C_60_ along a cartoon depicting the relative orientations of the two molecules in the face-on and edge-on bilayer samples; **b** Current-voltage characteristics of bilayer devices under 1 sun illumination

In this work, we begin by establishing that we are able to precisely control the molecular orientation of p-SIDT(FBTTh_2_)_2_^45^ (structure and orientations shown in Fig. 1a) in neat films, and fabricate bilayer heterojunctions with sharp, well-defined interfaces of known molecular orientations. We then analyze the photovoltaic performance, which reveals that molecular orientation has a profound effect on the *V*_OC_ and short-circuit current (*J*_SC_) (Fig. 1b). The higher *V*_OC_ of the solar cells with face-on p-SIDT(FBTTh_2_)_2_ is attributed to a higher CT state energy (*E*_CT_) and lower non-radiative recombination losses. However, the edge-on solar cells are more efficient at charge generation, illustrated by a higher internal quantum efficiency (IQE). Electronic-structure calculations predict that the face-on bilayers have a larger electronic coupling between the CT state and GS, suggesting that they suffer from greater geminate recombination. In addition, charge generation in face-on bilayers is significantly more temperature-dependent than edge-on bilayers, which may be a consequence of a larger barrier to charge generation or favorable polarization at the edge-on donor/acceptor interface. Our study presents important experimental evidence pertaining to the effect of molecular orientation on non-radiative recombination and the efficiency of charge generation.

## Results

### Structural characterization

**Fig. 2:**
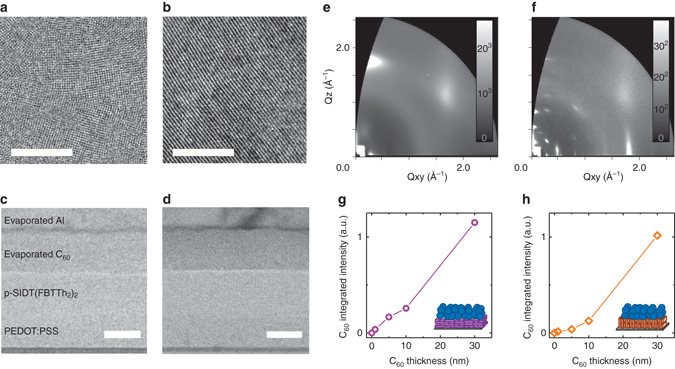
Structural characterization of the molecular orientation and interface quality. HR-TEM images (**a**, **b**) and GIWAXS spectra (**e**, **f**) of face-on (**a**, **e**) and edge-on (**b**, **f**) p-SIDT(FBTTh_2_)_2_ films. Cross-section HR-TEM of face-on (**c**) and edge-on (**d**) bilayers. Correlation of peak intensity fitting with C_60_ thickness evaporated on films of p-SIDT(FBTTh_2_)_2_ for face*-*on (**g**) and edge-on (**h**) samples. For more detail, refer to Supplementary Fig. 3. Scale bar for the HR-TEM images is 50 nm; scale bar for the cross-section HR-TEM images is 40 nm

To begin, films of neat p-SIDT(FBTTh_2_)_2_ were characterized to quantify bulk and interfacial molecular orientation. Preferential orientation can be measured using grazing incidence wide-angle X-ray scattering (GIWAXS), by comparing intensities of in-plane and out-of-plane *π*-stacking peaks (at *q* ~ 1.7 Å^−1^). More detail on the methodology is available in the Supplementary Fig. 1. When cast from chlorobenzene (CB), films of p-SIDT(FBTTh_2_)_2_ show a ratio of 99.5:0.5 face-on:edge-on orientation. When cast from CB with 0.4% v/v diiodooctane (DIO), films of p-SIDT(FBTTh_2_)_2_ show a ratio of 94:6 edge-on:face-on orientation. The two orientations also have distinctly different structures seen by high-resolution transmission electron microscope (HR-TEM) images. Figure 2a–d show HR-TEM and GIWAXS images of p-SIDT(FBTTh_2_)_2_ films cast from CB (face-on) and CB+DIO (edge-on).

In addition to providing information about the molecular orientation, the GIWAXS images in Figs. 2c, d also show that the edge-on p-SIDT(FBTTh_2_)_2_ films are more ordered than the face-on films. We do not believe that this difference in ordering has a large impact on the results discussed further in this study; we refer the reader to further data and discussion on this topic in Supplementary Fig. 2 and Supplementary Notes 1, 2.

The quality of the p-SIDT(FBTTh_2_)_2_ interface was verified by cross-section HR-TEM and GIWAXS. The miscibility of C_60_ into p-SIDT(FBTTh_2_)_2_ was determined by monitoring the GIWAXS signal of varying thicknesses of C_60_ evaporated on p-SIDT(FBTTh_2_)_2_ (Supplementary Fig. 3). A linear increase in C_60_ scattering intensity with C_60_ thickness indicates that C_60_ molecules are not diffusing into the p-SIDT(BFTTh_2_)_2_ layer beneath. Figure 2g shows a linear signal growth with C_60_ thickness for the face-on device, confirming that the interface is sharp. The trace for the edge-on device, Fig. 2h, shows a slight deviation from linearity at small thicknesses of C_60_. However, this can be explained by deposition of C_60_ into pinholes in the edge-on p-SIDT(FBTTh_2_)_2_ film which are not present in the face-on film (Supplementary Fig. 4). In addition to reducing the actual film thickness from the nominal predicted film thickness, the surface topography presented by pinholes may have an unpredictable effect on the X-ray scattering. Cross-section TEM (Figs. 2e, f) shows no evidence of interdiffusion for either face-on or edge-on bilayers. In fact, lattice planes can be well resolved in the cross-section TEM of the edge-on sample for the entire thickness of the p-SIDT(FBTTh_2_)_2_ layer (Fig. 2f). The lattice planes further confirm that the donor layers retain their orientation through the bulk of the film to the interface with C_60_, and that deposition of the C_60_ layer does not disrupt the packing of p-SIDT(FBTTh_2_)_2_. Thus, it can be concluded that the donor/acceptor interface is abrupt for edge-on and face-on p-SIDT(FBTTh_2_)_2_.

### Solar cell characteristics

The *J–V* characteristics of the edge-on and face-on bilayers, using identical contacts (ITO/PEDOT:PSS/active layer/BCP/Al, see Methods for full material names), under 1 sun illumination, are presented in Fig. 1b and Table 1. The *J*_SC_ and the fill factor (FF) are very similar for both molecular orientations of the donor layer. The *V*_OC_, on the other hand, is a substantial 150 mV larger when the donor molecules are face-on compared to edge-on.

**Table 1:** Solar cell characteristics of p-SIDT(FBTTh_2_)_2_/C_60_ devices with face-on or edge-on donor molecular orientation

	***V***_OC_ (**V**)	***J***_SC_ (mA/cm^2^)	**FF** (%)
Face-on	0.84 ± 0.03	−2.97 ± 0.3	66 ± 5
Edge-on	0.69 ± 0.04	−3.03 ± 0.4	68 ± 3

The solar cell characteristics here are averages of over 150 devices of each orientation; histograms for each parameter are available in Supplementary Fig. 5.

### Open circuit voltage

There are several factors that can account for the change in the *V*_OC_, and we will explore each in turn: IP and *E*_CT_, radiative recombination, and non-radiative recombination.

A number of studies have demonstrated that changing molecular orientation in a film can lead to differences in the material energy levels^7–9, 11, 16, 17^, which can have a direct effect on the *V*_OC_. Indeed, ultraviolet photoelectron spectroscopy (UPS) measurements of neat p-SIDT(FBTTh_2_)_2_ films show an increase on the order of 60 meV in the IP of p-SIDT(FBTTh_2_)_2_ when it is face-on (Supplementary Fig. 6). In good agreement, electronic-structure calculations performed on p-SIDT(FBTTh_2_)_2_ also found that the face-on orientation has a deeper ionization potential (Supplementary Fig. 7). However, as the *V*_OC_ varies by 150 mV, changing the molecular orientation has altered more than just the IP value.

It has been demonstrated numerous times that *E*_CT_ and *V*_OC_ tend to correlate according to: *E*_CT_−*qV*_OC_ = 0.6 ± 0.1 eV^23, 46^. The CT state is routinely studied with highly sensitive absorption techniques (here, we use external quantum efficiency, EQE), where it is identified as a shoulder at sub-bandgap energies^46^. The CT state can also be studied by emission spectra (here we use electroluminescence, EL), where it is identified as a featureless emission spectrum at low energies^47^. The *E*_CT_, located at the midpoint between the absorption and emission of the CT state, can be determined by a simultaneous fit to the measured absorption (Eq. 1) and emission spectra (Eq. 2)^48^:

1 $$\sigma \left( E \right)E = \frac{f}{{\sqrt {4\pi \lambda kT} }}{\rm exp}\left( {\frac{{ - {{({E_{\rm CT}} + \lambda - E)}^2}}}{{4\lambda kT}}} \right)$$

2 $$\frac{{I\left( E \right)}}{E} = \frac{f}{{\sqrt {4\pi \lambda kT} }}{\rm exp}\left( {\frac{{ - {{({E_{\rm CT}} - \lambda - E)}^2}}}{{4\lambda kT}}} \right)$$

In these equations, *k* denotes Boltzmann’s constant; *T*, temperature; and *E*, photon energy. The fit parameters are *E*_CT_ (energy of the CT state), *λ* (reorganization energy), and *f* (a parameter proportional to the number of CT states and the square of their coupling matrix element with the GS). Eqs. 1 and 2 are called the reduced absorption and emission spectra, due to the multiplication and division of the spectra by *E*, respectively.

**Fig. 3:**
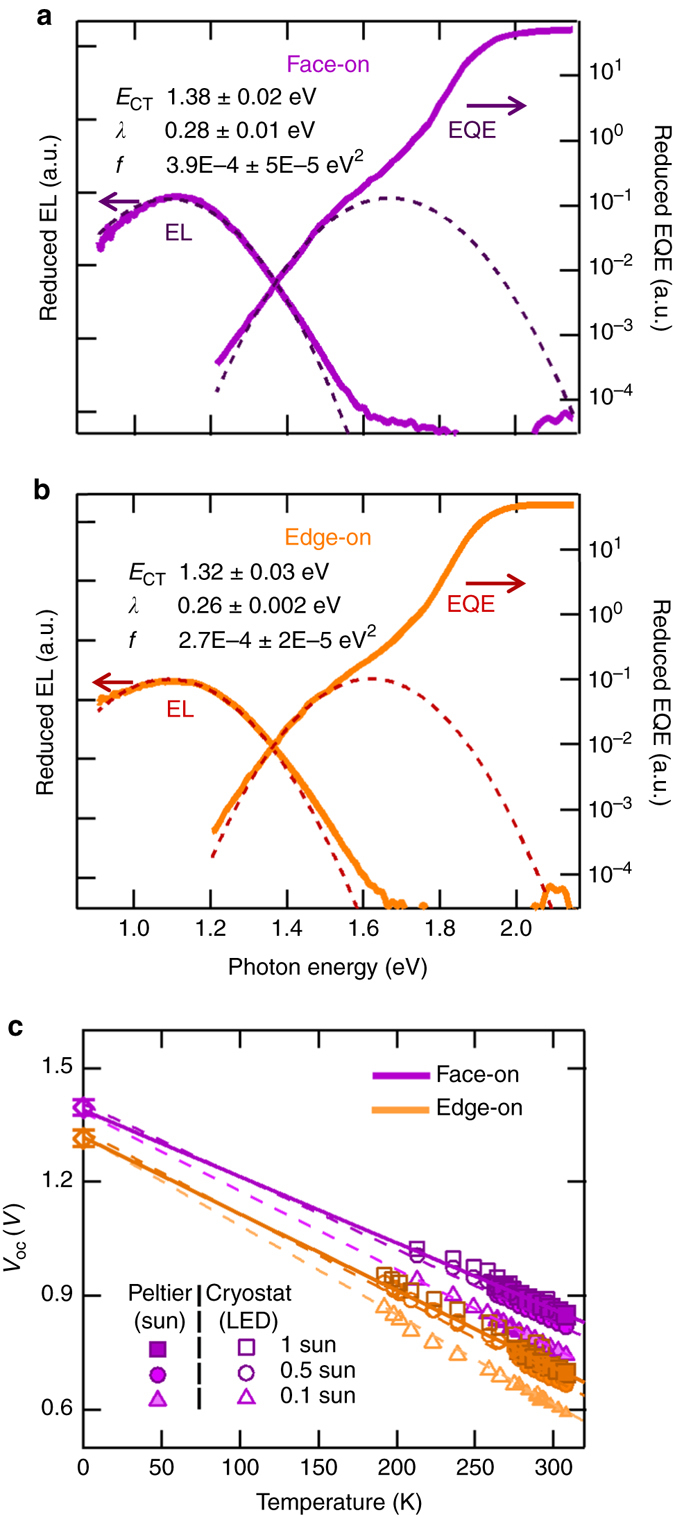
Characterization of the CT state in the bilayers. **a**, **b** EQE spectra of the sub-bandgap absorption and the corresponding EL spectra for face-on (**a**) and edge-on (**b**) bilayers. *Dashed lines* are fits to the EQE using Eqs. 1 and 2. Fit parameters and their respective s.d. errors are reported in the figures. **c** Temperature-dependent *V*_OC_ at 1, 0.5, and 0.1 suns, extrapolated to 0 K represented on the axis with the corresponding standard deviations

Using Eqs. 1 and 2, fit simultaneously to the EQE and EL spectra, we obtain that *E*_CT_ is 1.38 ± 0.02 eV when p-SIDT(FBTTh_2_)_2_ is face-on vs. 1.32 ± 0.03 eV for the edge-on orientation. The EQE, EL and their corresponding fits are shown in Fig. 3a–b. The 60 meV higher *E*_CT_ is in excellent agreement with the higher ionization energy of the face-on p-SIDT(FBTTh_2_)_2_ layer, implying vacuum alignment at the interface to C_60_. However, it does not explain the full difference in *V*_OC_ upon changing molecular orientation.

Another estimate for the *E*_CT_ can be obtained by temperature-dependent *V*_OC_ measurements extrapolated to 0 K^48, 49^, where the deviation from *E*_CT_ to *V*_OC_ at room temperature (RT) should correlate with losses in the solar cell. One model that has been demonstrated on a number of systems separates the losses from *E*_CT_ into radiative and non-radiative recombination, shown in Eq. 3 ^48^:

3 $${V_{{\rm{OC}}}} = \frac{{{E_{{\rm{CT}}}}}}{q} - {\Delta V_{{\rm{rad}}}}\left( T \right) - {\rm{\Delta }}{V_{{\rm{nonrad}}}}\left( T \right)$$

with ${\Delta V_{{\rm{rad}}}}$(*T*) and ${\Delta V_{{\rm{nonrad}}}}$(*T*) the temperature-dependent radiative and non-radiative recombination losses given by Eqs. 4 and 5:

4 $${\Delta V_{{\rm{rad}}}}\left( T \right) = - \frac{{kT}}{q}{\rm ln}\left( {\frac{{{J_{{\rm{SC}}}}{h^3}{c^2}}}{{fq2\pi \left( {{E_{{\rm{CT}}}} - \lambda } \right)}}} \right)$$

5 $${\rm{\Delta }}{V_{{\rm{nonrad}}}}\left( T \right) = - \frac{{kT}}{q} \rm ln\left( {EQ{E_{{\rm{EL}}}}} \right)$$

In these equations, *J*_SC_ represents the short circuit current; *h*, Planck’s constant; *c*, speed of light; *E*_CT_, *f*, *λ* are fit parameters from Eqs. 1 and 2 (Fig. 3a, b); and EQE_EL_ is the total external quantum efficiency of electroluminescence.

The *V*_OC_ values of each bilayer were measured at different light intensities and temperatures ranging from 190 to 310 K, and extrapolated to 0 K, as shown in Fig. 3c. For all light intensities the *V*_OC_ extrapolated to 0 K is 1.40 ± 0.02 V for the face-on compared to 1.31 ± 0.02 V for the edge-on solar cells, which is in close agreement to the *E*_CT_ values obtained from the fits of the EQE and EL spectra. This confirms our interpretation from above that the difference in *V*_OC_ is not solely due to energetics. In fact, from the temperature-dependent slopes in Fig. 3c, it is evident that the overall *V*_OC_ loss is smaller in face-on than in edge-on bilayers.

As shown by Eq. 3, the voltage loss can be quantified into radiative and non-radiative recombination contributions, as has been outlined by Rau^31^ and Vandewal et al.^48^ Radiative recombination can be estimated according to Eq. 4. We find that at room temperature Δ*V*_rad_ is very similar, 199 ± 2 mV and 197 ± 4 mV for the face-on and edge-on bilayers, respectively, leading to the conclusion that losses due to radiative recombination are not a function of molecular orientation.

Non-radiative recombination remains poorly understood, yet it is considered to be among the primary reasons that the overall *V*_OC_ loss has not decreased significantly in recent years^25^. Most organic blends reported in the literature lose 300–400 mV to non-radiative recombination, constituting 60% or more of the lost potential^25, 48^. To estimate the effect of orientation on non-radiative recombination, we refer to EQE_EL_, defined as photons emitted per electrons injected into the device. The lower the radiative efficiency, the more non-radiative decay channels contribute to the overall recombination. Equation 5 relates EQE_EL_ to the voltage loss.

Importantly, the measured EQE_EL_ values differ by more than an order of magnitude between the two samples, with 3.2 × 10^−6^ ± 1.4 × 10^−6^ for the face-on and 2.3 × 10^−7^ ± 3.5 × 10^−7^ for the edge-on bilayers. Using Eq. 5, we find that voltage losses due to non-radiative recombination are 316 ± 10 mV and 382 ± 28 mV for the face-on and edge-on solar cells, respectively.

Table 2 summarizes the energetic and recombination differences between the bilayers with the two orientations. The estimated difference in *V*_OC_ between the two orientations, *V*_OC,calc_, calculated from the measured *E*_CT_ and the estimated losses due to radiative and non-radiative recombinations (Eq. 3) is in very good agreement with the measured Δ*V*_OC_, determined experimentally from the *J–V* characteristics.

**Table 2:** Summary of ***E***_CT_ and recombination voltage losses for the bilayer solar cells

	***E***_CT_ (eV)	**Radiative loss (mV)**	**Non-radiative loss (mV)**	***V***_OC,calc_ (V)	***V***_OC_ (V)
Face-on	1.38 ± 0.02	199 ± 2	316 ± 10	0.87	0.84 ± 0.03
Edge-on	1.32 ± 0.03	197 ± 4	382 ± 28	0.74	0.69 ± 0.04

The significance of these findings lies in the differences in non-radiative recombination: on average, the edge-on solar cells lose 66 mV more voltage than face-on cells due to non-radiative recombination. Using the same system but flipping the donor molecular orientation, the non-radiative recombination pathway has been altered, implying it is sensitive to the molecular alignment at the donor/acceptor interface. Interestingly, these experimental results are fully consistent with a recent theoretical study: Chen et al.^3^ found that the higher *E*_CT_ value and greater hole delocalization and migration away from a face-on pentacene/C_60_ interface caused a decrease in vibronic coupling of the CT state to the GS, thus reducing the non-radiative recombination rate. While the results by Chen et al.^3^ refer to model molecular packings of pentacene, the similarity to our findings on p-SIDT(FBTTh_2_)_2_/C_60_, namely a smaller *E*_CT_ and more non-radiative recombination in the edge-on bilayers, is striking.

### Short circuit current

In contrast to the *V*_OC_, the *J*_SC_ appears independent of molecular orientation (Fig. 1). However, due to alignment of the molecular transition dipoles, the absorption strength of the two bilayers toward normal incident light is significantly different: face-on p-SIDT(FBTTh_2_)_2_ films have a two times higher absorbance as edge-on films (Supplementary Fig. 8). Using a combination of Monte Carlo (MC) and molecular dynamics (MD) simulations, we find that p-SIDT(FBTTh_2_)_2_ has a unique unit cell, shown in Supplementary Fig. 9, true for both molecular orientations. As a consequence, half the p-SIDT(FBTTh_2_)_2_ molecules in the edge-on orientation are lying down with their long edge parallel to the substrate, and half the molecules are standing up on their end, with their sides perpendicular to the substrate (as depicted in Fig. 1a). In the face-on orientation, all the p-SIDT(FBTTh_2_)_2_ molecules are parallel to the substrate. This model for the packing of p-SIDT(FBTTh_2_)_2_ is in good agreement with the two-times higher absorbance of the face-on films. Furthermore, to verify that the results we have reported thus far are not a consequence of the different geometry of molecular transition dipoles with respect to light, we measured the current-voltage (*J-V*) curves for the bilayer solar cells as a function of illumination angle. The *V*_OC_ remains unchanged, while the *J*_SC_ increases by a small amount (Supplementary Fig. 10).

**Fig. 4:**
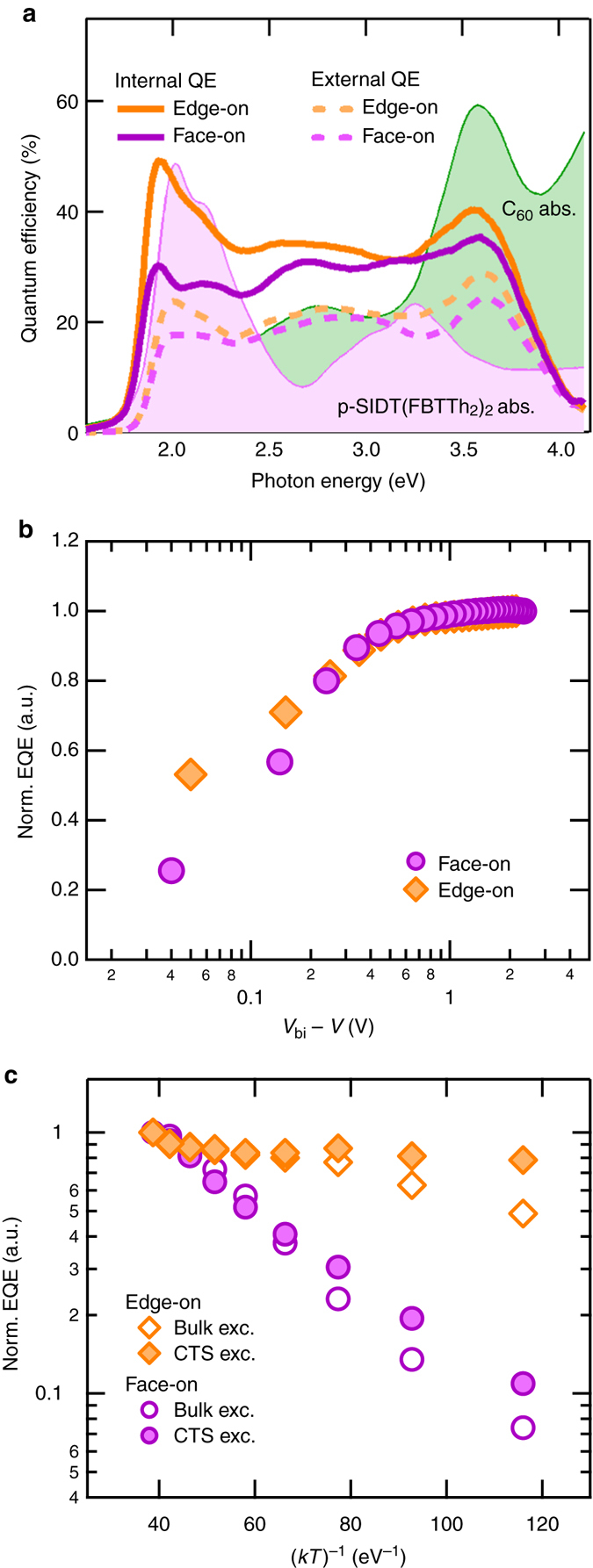
Efficiency of charge generation. **a** EQE (*dashed lines*) and IQE (*continuous lines*) spectra of bilayer solar cells with varying orientation. The absorption spectra of p-SIDT(FBTTh_2_)_2_ and C_60_ are plotted in the background for reference. **b** Bias-dependent EQE values corresponding to p-SIDT(FBTTh_2_)_2_ absorption, integrated and normalized to the value under the highest field. The applied bias was corrected for the built-in potential. **c** Temperature-dependent EQE values, integrated and normalized to the value at room temperature. Open symbols are EQE values integrated over bulk absorption, and full symbols are EQE values integrated only for CT state absorption

The similar *J*_SC_ values are in agreement with the EQE (quantum efficiency per incident photon) of the bilayers, both peaking at about 25% on average. However, when the EQE spectra are corrected for absorption of the active layer (device absorption corrected for parasitic absorption, more detail in Supplementary Fig. 11), we obtain quantum efficiency spectra per absorbed photons, i.e., IQE. EQE and IQE spectra of the bilayers are shown in Fig. 4a. For reference, the unitless absorption spectra of p-SIDT(FBTTh_2_)_2_ and C_60_ are shown in the background.

IQE of the edge-on bilayers is higher than the face-on bilayers (Fig. 4a). A higher IQE indicates that in edge-on bilayers fewer excitons and charges recombine at short circuit. In other words, the edge-on solar cells have more efficient charge generation. Charge generation depends on exciton quenching efficiency and geminate recombination. Photoluminescence (PL) quenching measurements comparing neat edge-on and face-on films with their respective bilayer structure show that both molecular orientations have the same quenching efficiency (Supplementary Fig. 12). Therefore, the difference in the IQE values must originate from geminate recombination after charge transfer across the heterojunction. This finding is consistent with the results of our electronic-structure calculations, which indicate that electronic coupling between CT state and GS is weaker in the edge-on configuration than in the face-on configuration (Supplementary Fig. 13). A smaller electronic coupling is expected to decrease the rate of geminate recombination as the CT state tends to dissociate, shifting the generation-recombination balance towards free charge formation, as seen here^2^.

For a deeper understanding of the differences in IQE, we measured the EQE under varying electric fields and temperatures. These measurements are complex, since they reflect the combination of many processes such as exciton diffusion, charge transfer, charge generation, bimolecular recombination, and charge transport. However, with the appropriate conditions and analysis, EQE measurements can be used to gain insight into charge generation/geminate recombination. Specifically, to eliminate effects of exciton diffusion and charge transfer, EQE spectra can be analyzed at energies corresponding to CT state absorption. Also, since these devices are bilayers and the measurements are carried out at low light intensities and under an internal field (*J*_SC_ conditions, unless otherwise stated), bimolecular recombination and the effect of differences in of charge transport are expected to be negligible. Under these conditions, our EQE measurements should reflect the dependence of charge generation/geminate recombination on electric field and temperature.

First, we asked if changing interfacial molecular orientation has an impact on the Coulomb binding energy of the CT state. The binding energy, a consequence of the electrostatic attraction between opposite charge carriers, depends on the electron-hole separation and the dielectric constant^50^. The binding energy can be overcome with the assistance of a field^40, 51^, and thus it may follow that the field-dependence of generation would be different for the two bilayers^52^. Figure 4b shows the effect of an electric field on the EQE of the devices: both have a very similar dependence on the electric field. The EQE values in Fig. 4b are normalized to the efficiency at the strongest applied bias, and the field across the device is corrected by the built-in voltage, analogous to photocurrent analysis (for bias-dependent EQE spectra, see Supplementary Fig. 14). Time delayed collection field (TDCF) measurements confirm no significant differences in the field dependence of generation between the two orientations (Supplementary Fig. 15). Furthermore, by TDCF there is no difference in field-dependence of generation for excitations at 350 nm, 600 nm, or 650 nm, ruling out any effects of hot-exciton generation. All these results indicate that the binding energy of the CT state is not a function of molecular orientation.

Next, we turned our attention to the temperature-dependence of charge generation. Figure 4c shows EQE values normalized to the EQE at RT, as a function of temperature (for temperature-dependent EQE spectra, see Supplementary Fig. 16). The temperature dependence is shown for absorption over all energies, as well as absorption corresponding only to the CT state (~1.2–1.5 eV). Overall, while charge generation in both bilayers is temperature-dependent, the temperature-dependence in the face-on bilayers is much stronger, indicating a larger activation energy for charge generation. To illustrate this point, if we make crude simplifications and extrapolate the EQE values to the limit of temperature→0 K, we find that generation in the face-on bilayer becomes negligible, while the edge-on bilayer can still generate about 10–40% carriers (as compared to carrier generation at RT). Charge generation resulting from bulk and direct CT state excitations follows similar temperature dependences, as a function of p-SIDT(FBTTh_2_)_2_ orientation. The contrast in temperature dependence is therefore not due to differences in exciton diffusion or electron transfer, but is instead a result of difference in charge generation with interfacial molecular orientation. This can be explained by a larger barrier to charge generation in the face-on bilayer due to elements such as electronic coupling between the CT states and separated states or polarization at the donor/acceptor interface.

## Discussion

In conclusion, we have been able to fabricate donor-acceptor bilayers with sharp interfaces and well-defined p-SIDT(FBTTh_2_)_2_ molecular orientations: either face-on or edge-on with respect to the substrate. These orientations are preserved through the donor film to the interface with C_60_, with none-to-minimal diffusion at the donor/acceptor interface. This unprecedented precise morphological control reveals the genuine effects of molecular orientation on photovoltaic performance. Edge-on p-SIDT(FBTTh_2_)_2_ bilayers suffer from greater non-radiative recombination and a reduced *E*_CT_, which result in a substantial *V*_OC_ loss of 150 mV. However, charge generation is more efficient when the donor/acceptor interface is edge-on, evidenced by a higher IQE. This is attributed to reduced CT state-GS electronic coupling as well as smaller activation energy for charge generation in the edge-on bilayer, which may be a consequence of a reduced barrier between CT state and charge-separated states or favorable polarization at the donor/acceptor interface.

The lessons learned from p-SIDT(FBTTh_2_)_2_/C_60_ bilayers can be summarized into two major points: First, in applications which benefit from high radiative efficiency (such as LEDs and OPVs), interfacial molecular orientation should reduce non-radiative recombination (by reducing charge recombination through triplet states or through vibronic coupling of the CT state to the GS). Our results establish that face-on molecular orientations would achieve higher radiative efficiency. Second, In applications where charge separation is important (such as photodetectors and OPVs), the electronic couplings between the CT state and the GS, as well as the activation energy for charge generation should both be minimized. This can perhaps be accomplished by beneficial polarization effects. Thus, our results determine that interfacial molecular orientation should be edge-on for intrinsic, efficient charge generation.

Overall, in the case of OPV, these two lessons go in opposite directions. These results highlight that to achieve high performance in OPV, the electronic coupling for face-on donor/acceptor interactions must be reduced to eliminate geminate recombination. Conversely, more research on non-radiative recombination is necessary in order to curtail the resulting losses and benefit from the improved charge generation in an edge-on donor/acceptor interaction. It may be possible to tackle both problems from the perspectives of molecular design and clever device engineering.

## Methods

### Sample preparation

p-SIDT(FBTTh_2_)_2_ (benzo[1,2-*b*:4,5-*b*]bis(4,4′-dihexyl-4*H*-silolo[3,2-*b*]thiophene-2,2′-diyl)bis(6-fluoro-4-(5′-hexyl-[2,2′-bithiophene]-5-yl)benzo[*c*][1,2,5]thiadiazole) was synthesized according to the previously reported scheme^45^. p-SIDT(FBTTh_2_)_2_ was dissolved at a concentration of 15 mg ml^−1^ in pristine CB, or CB with 0.4% DIO v/v. In the CB+DIO solution, the conditions correspond to a ratio of two molecules DIO for every one molecule of p-SIDT(FBTTh_2_)_2_^53^. The face-on films used in this study were achieved by spin-casting p-SIDT(FBTTh_2_)_2_ from pristine CB solutions, and the edge-on films were achieved by casting from CB+DIO solutions, at 2000 round per minute (RPM). Bilayer devices were fabricated on glass substrates sputtered with ITO, and coated with 35 nm poly(3,4-ehtylenedioxythiophene):poly(styrenesulfonate) (PEDOT:PSS). A C_60_ layer was thermally evaporated on p-SIDT(FBTTh_2_)_2_, followed by bathocuproine (BCP) and aluminum (Al). The final device structure for nearly all measurements reported herein is as follows: ITO/PEDOT (35 nm)/p-SIDT(FBTTh_2_)_2_ (45 nm)/C_60_ (45 nm)/BCP (4 nm)/Al (80 nm).

### GIWAXS analysis

GIWAXS was performed at Stanford Synchrotron Radiation Lightsource beamline 11-3 with a MAR345 image plate. The data was calibrated and reduced using WxDiff software package^54^. To characterize the amount of edge-on vs. face-on material, a cake slice around the *π*-stacking peak, between *Q* = 1.8 Å^−1^ and 2.0 Å^−1^, was reduced to a pole figure. An adjacent cake slice between *Q* = 1.7 Å^−1^ and 1.8 Å^−1^ was subtracted from the data to account for background scattering (see Supplementary Fig. 1). The data was fit to two in-plane *π*-stacking peaks at about −90° and +90°, one out-of-plane *π*-stacking peak at 0°, and four peaks around ±30° and ±55° representing the SiO_2_ substrate background. The out-of-plane peak area was compared to the average of the two in-plane peak areas to arrive at the face-on to edge-on ratio for each sample. Samples for GIWAXS were prepared on cleaned SiO_2_ substrates, coated with PEDOT:PSS and p-SIDT(FBTTh_2_)_2_ cast from 15 mg ml^−1^ CB or CB+DIO.

The C_60_ scattering as a function of C_60_ thickness was tracked by reducing cake slices shown in Supplementary Fig. 3 to *I* vs. *Q*. We selected these specific cake slices for analysis because they contain non-overlapping peaks from both the small molecule and the C_60_, allowing a simultaneous comparison of contributions from both materials. This data was fit to a linear combination of neat p-SIDT(FBTTh_2_)_2_ and neat C_60_ data, and the fit coefficient for C_60_ was reported. Fits are shown in Supplementary Fig. 3 along with the neat data. Fits have some discrepancy due to small changes in peak shape but are reasonably accurate in depicting overall trends. Samples for these measurements were prepared on cleaned SiO_2_ substrates, coated with PEDOT:PSS and p-SIDT(FBTTh_2_)_2_ cast from 15 mg ml^−1^ CB or CB+DIO, with C_60_ evaporated for thicknesses of 0–30 nm.

### Ultraviolet photoelectron spectroscopy

UPS samples were prepared on ITO-sputtered glass substrates cleaned by sonication in water, acetone, and isopropyl alcohol for 15 min, followed by UVO treatment for 1 h. ITO pieces were immediately transferred to the glove box. Solutions of 7 mg ml^−1^ p-SIDT(FBTTh_2_)_2_ in CB or CB+0.4% DIO were prepared ahead of time, and allowed to stir on the hot plate at 110 °C. Prior to casting the films, the solutions were brought to room temperature. Films were cast at 4000 RPM for film thicknesses of about 10 nm; thicker films of p-SIDT(FBTTh_2_)_2_ led to distortion of the UPS signal due to charging in the film. The UPS samples were transferred to an ultrahigh vacuum chamber from the nitrogen glove box in an airtight chamber to avoid any surface degradation or contamination with air. The UPS was measured with a HeI source (21.2 eV), at pressures of low 10^−7^ Pa, a constant pass of 5 eV, current of 10 mA, and the anode set to 6 kV. The ionization potential was calculated according to *I*_p_ *=* 21.2 eV−(*E*_cutoff_ *−E*_g_)^55^, where the *E*_cutoff_ and *E*_g_ were determined by the onset at high and low binding energies, respectively. The energy resolution of the UPS measurements is below 10 meV.

### *J–V* characteristics

Solar-cell device properties were measured under illumination by a simulated 100 mW cm^−2^ AM1.5 G light source using a 300 W Xe arc lamp with an AM 1.5 global filter. The irradiance was adjusted to one sun with a standard silicon photovoltaic calibrated by the National Renewable Energy Laboratory. Temperature dependent *V*_OC_ in the range of 275–300 K was collected using a custom Peltier cooled sample holder under illumination from the above described light source in combination with optical density filters to reduce the intensity. In the range of temperatures below 275 K, a helium cryostat was used for temperature control and illumination was achieved with a high power 1 W, 445 nm laser diode (fluence was tuned with the DC bias applied to the laser diode to match light intensity used in the solar simulator). Bias dependent *J–V* spectra were measured by changing the angle of the light source, while keeping the device constant. Before each *J–V* measurement light intensity was calibrated to 1-sun using the calibrated silicon photovoltaic.

### TEM and cross-sectional TEM

TEM samples were prepared by casting a layer of p-SIDT(FBTTh_2_)_2_ from CB or CB+DIO on PEDOT:PSS, and floating pieces of the film on deionized (DI) water. Film pieces were transferred to TEM grids and allowed to dry overnight. High-resolution images were taken with an FEI Titan FEG High Resolution microscope. The TEM images were collected using a low-dose electron beam (spot size 6) to avoid beam damage, and a small defocus to enhance the contrast in the images.

Using an FEI focused ion-beam (FIB) microscope, a 20 µm long slice with a thickness of about 200 nm was cut from a bilayer device (prepared as described above), and mounted on a TEM grid. The donor/acceptor interface in the bilayers was then imaged by HR-TEM. The procedure followed has been described in detail previously^56, 57^. Careful attention was devoted to minimize exposure of the sample to high-energy electron and ion beams, thereby reducing damage as much as possible.

### Atomic force microscopy

Atomic force microscopy (AFM) images were collected in air, using a Si tip, and an Innova AFM operated under tapping mode.

### UV-visible absorption spectroscopy

Absorption spectra of neat face-on and edge-on p-SIDT(FBTTh_2_)_2_ and C_60_ films were collected using a Perkin Elmer Lambda 750 spectrophotometer. Samples for absorption measurements were prepared on clean glass slides, following the same procedure used to prepare the respective layer in the bilayer solar cell devices: face-on and edge-on p-SIDT(FBTTh_2_)_2_ films were solution cast from their respective solutions, and C_60_ films were thermally evaporated. The film thickness for all samples was 45 nm.

### External and internal quantum efficiency

EQE characteristics were measured in a nitrogen-filled glovebox using a setup at UCSB and in Potsdam University. At UCSB, the EQE setup consisted of a 75 W Xe light source, monochromator, optical chopper, lock-in amplifier, and a National Institute of Standards and Technology calibrated silicon photodiode for power-density calibration. At Potsdam University, the EQE setup is similar, but used a 200 W halogen lamp (Philips), and a UV enhanced silicon photodiode to calibrate the visible spectra, or a germanium photodiode to calibrate the near infrared spectra. Both photodiodes were calibrated by Newport. For the sub-bandgap EQE, higher sensitivity settings were used with a longer time delay between measurement points. Bias-dependent EQE was collected on the setup in Potsdam University, coupled to a Keithley source-measure-unit used to apply a bias while the EQE spectra were recorded. Temperature-dependent EQE measurements were collected with a setup at UCSB, following a similar procedure, using a nitrogen-cooled cryostat.

Total absorption of solar cell devices was measured with an integrating sphere, and corrected for parasitic absorption as determined for the bilayers using a transfer matrix model^58^. Subtracting the parasitic absorption from the total device absorption then gives the active layer absorption, and dividing EQE spectra by the corresponding active layer absorption gives the IQE spectra of the device.

### EL and its efficiency

EL spectra for the bilayers were collected directly from the solar cell devices, by applying a bias that is close to the turn-on voltage of the devices. The resulting emission was collected with an Andor SR393i-B spectrometer provided with a cooled silicon detector DU420ABR-DD and a cooled InGaAs DU491A-1.7 detector. The spectra were corrected for detector response using a blackbody spectrum.

The EL efficiency was collected by applying a small bias to the bilayer devices, and placing a calibrated silicon photodiode directly in front of the device to collect the resulting emission. The angle between the calibrated silicon photodiode and the device was varied to account for anisotropy in emission intensity.

### PL quenching measurements

PL samples were prepared by spin-coating p-SIDT(FBTTh_2_)_2_ films (face-on or edge-on) on clean glass slides, and evaporating C_60_ on half the slides, analogous to the preparation of the active layer in the bilayer devices. The samples were encapsulated inside the glovebox by applying epoxy around the edges of the glass slide and putting another glass slide on top, to create an air-tight, nitrogen environment. The samples were excited by a laser excitation at 455 nm, and the PL collected using a silicon CCD camera, cooled to −70 °C. A series of lenses were used to collect the PL and focus it on the CCD camera slit. The resulting spectra were corrected for instrument response by using a reference blackbody spectrum.

### Time-delayed collection field measurements

Excitation is realized by a laser system consisting of a Libra USP-1K-HE with a pulse energy of ~4.0 mJ at 1 kHz and an OPerA Solo for wavelength selection. The pre- and collection voltage is applied via an Agilent 81150A pulse generator in combination with a home-built amplifier. Currents through the devices are measured via a 50 Ω resistor and recorded with an Yokogawa DL9140 oscilloscope.

### Electronic-structure calculations

The p-SIDT(FBTTh_2_)_2_ bulk structure and C_60_/p-SIDT(FBTTh_2_)_2_ bilayer structure were generated by a combination of MC and MD simulations. The MD simulations were run for 200 ps at 300 K with under the NVT ensemble using the Verlet integrator with a time step of 1 fs. The temperature was maintained by the Nose-Hoover thermostat. A spherical cutoff of 1.25 nm for the summation of van der Waals interactions and the Ewald solver for long-range Coulomb interactions was used throughout. The COMPASS force field as implemented in the Forcite program of Materials Studio was used for the MD simulations^59^. Density-functional theory calculations using the range-separated HSE functional were then carried out for face-on and edge-on p-SIDT(FBTTh_2_)_2_ slabs under periodic boundary conditions using the plane-wave based Vienna Ab-initio Simulation Package^60–63^.

The electron couplings between diabatic CT state and the GS of the p-SIDT(FBTTh_2_)_2_/C_60_ complexes were evaluated by means of the generalized Mulliken-Hush approach at the MD generated geometry. These calculations were performed with the B3LYP functional and 6–31G(d,p) basis set, using the Q-Chem package^64^.

### Data availability

Data and materials necessary to replicate this work are available upon request from the authors.

## Electronic supplementary material

Supplementary Information
