# Supplementary material for: Impact of interfacial molecular orientation on radiative recombination and charge generation efficiency
Source: Nat Commun. 2017 Jul 19;8:79. doi: 10.1038/s41467-017-00107-4 (PMC5517510; doi:10.1038/s41467-017-00107-4)
Supplement: Supplementary file 1 — Supplementary Information [file 41467_2017_107_MOESM1_ESM.pdf]

File name: Supplementary Information
Description: Supplementary figures, supplementary notes and supplementary references.

Supplementary Figures

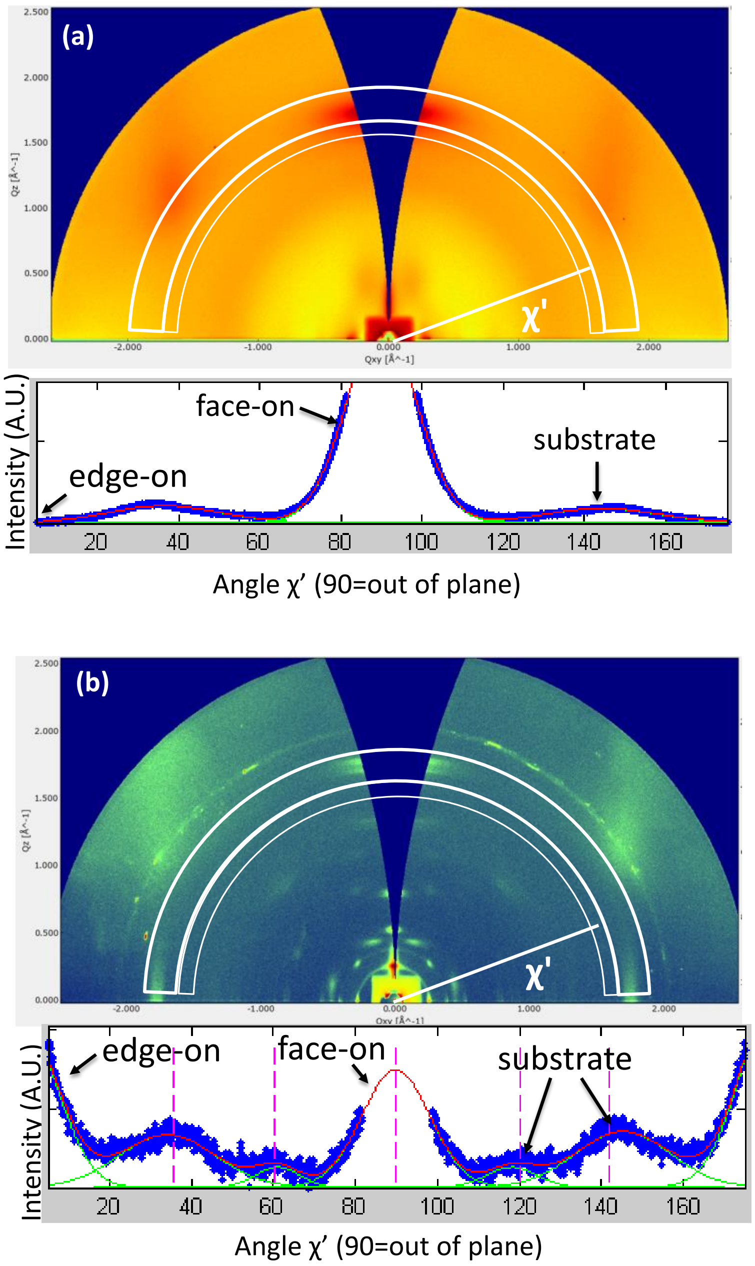

Supplementary Figure 1. Quantifying the molecular orientation in p-SIDT(FBTTh$_2$)$_2$ films. GIWAXS of p-SIDT(FBTTh$_2$)$_2$ cast from chlorobenzene (a) or from chlorobenzene with 0.4% w/w diiodooctane (b). The amount of face-on vs. edge-on charcter is calculated from the anistropy of the π-stacking peak, as a function of the polar angle, χ, from the substrate plane. The intensity must be corrected for solid angle effects using: intensity×|sin(χ-90)|.[1] While the signal from the GIWAXS scans originates only from the ordered portions of the films, both face-on and edge-on films are thin (45 nm) and significantly crystalline, justifying orientation assignment for the bulk films by crystalline scattering.

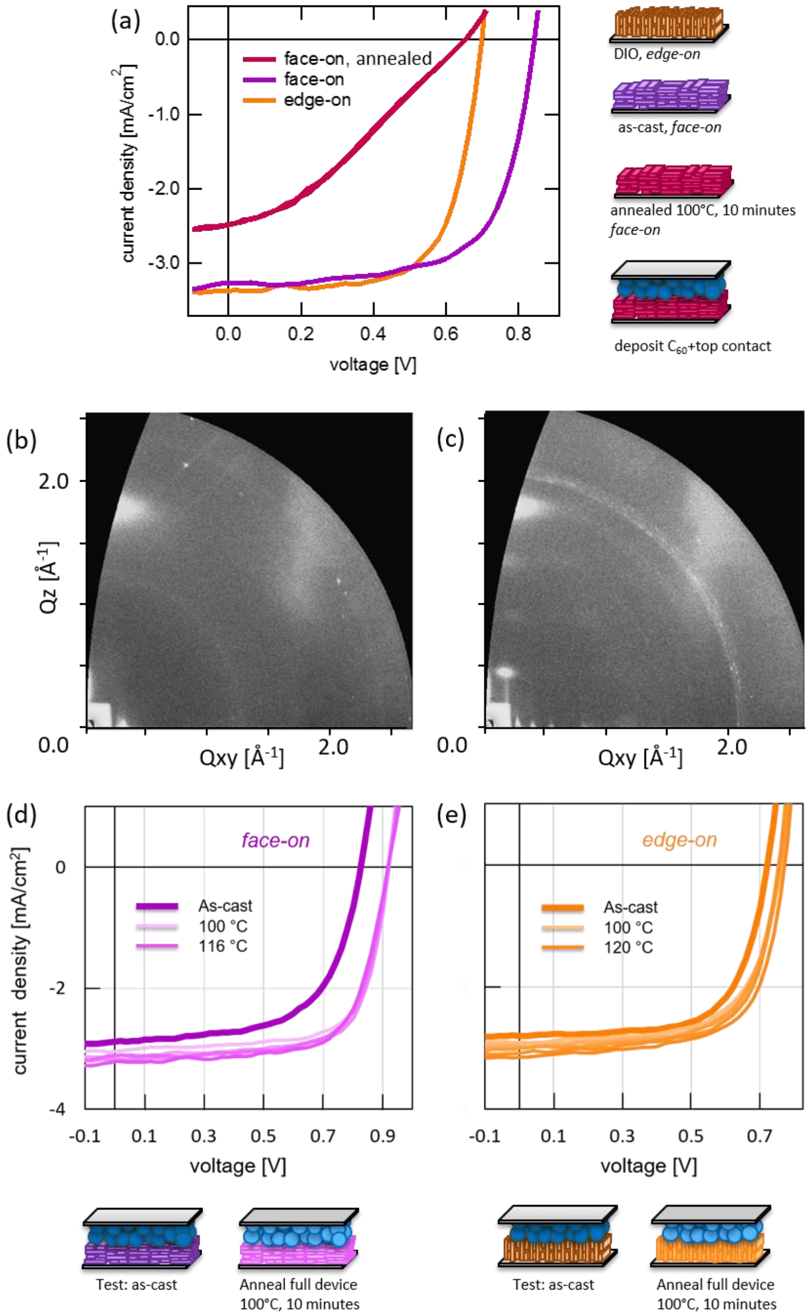

Supplementary Figure 2. Effect of annealing p-SIDT(FBTTh$_2$)$_2$ films and full bilayer devices. The effect of annealing p-SIDT(FBTTh$_2$)$_2$ films before deposition of C_{60} and the top-contact is shown in (a). GIWAXS for (b) as-cast face-on p-SIDT(FBTTh$_2$)$_2$ and (c) annealed face-on (100°C for 10 minutes) p-SIDT(FBTTh$_2$)$_2$ films. The effect of annealing the full bilayer devices (ITO/PEDOT/p-SIDT(FBTTh$_2$)$_2$/C_{60}/BCP/Al) at 100°C for 10 minutes is shown as *J*-*V* curves under 1-sun illumination for the face-on (d) and edge-on (e) devices.

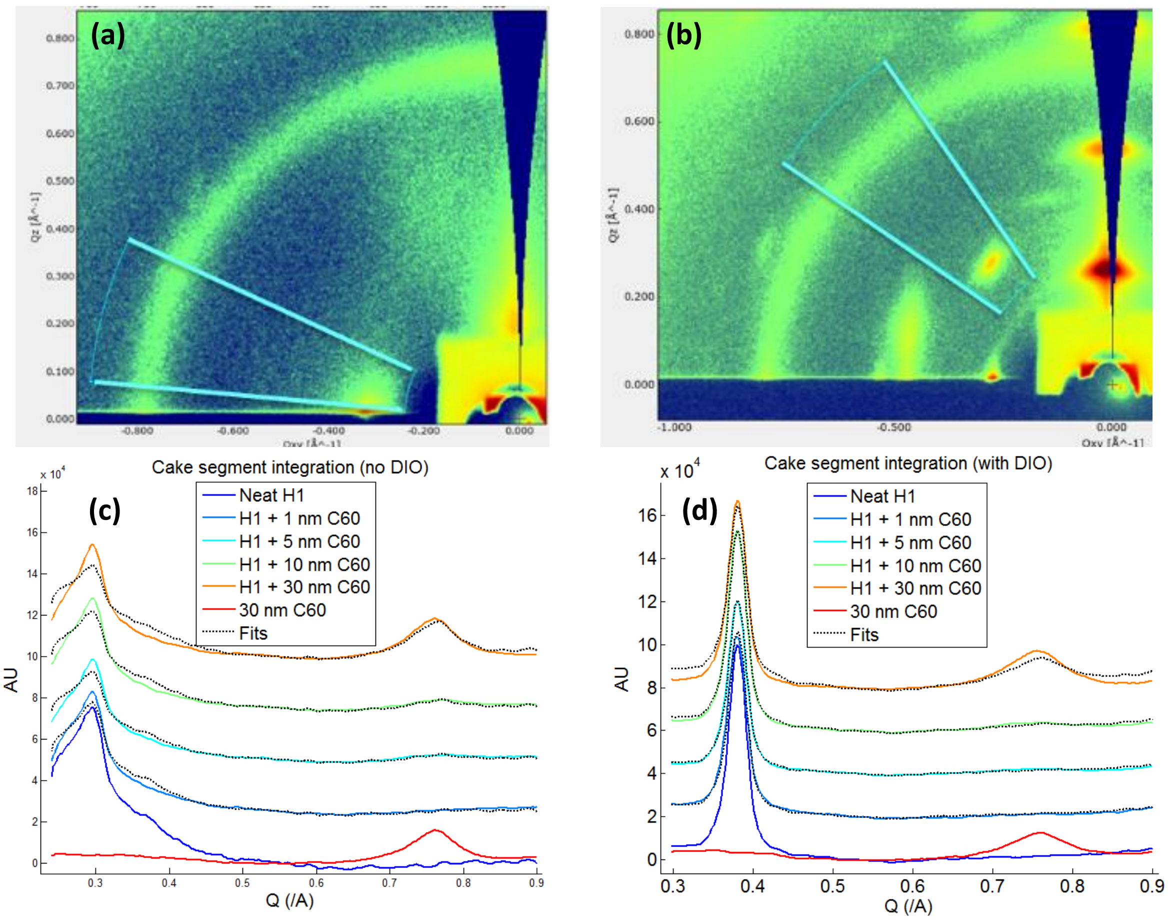

Supplementary Figure 3. Tracking interfacial mixing for C_{60}/p-SIDT(FBTTh$_2$)$_2$ interface. GIWAXS of face-on (a) and edge-on (b) p-SIDT(FBTTh2$_2$)$_2$ with C_{60} evaporated on top, showing signal for p-SIDT(FBTTh2$_2$)$_2$ and C_{60}. The cake slices in (a) and (b) show where the signal for p-SIDT(FBTTh2$_2$)$_2$ and C_{60} was fit for the two samples. (c,d) show the scattered intensity for face-on and edge-on p-SIDT(FBTTh-2$_2$)$_2$, respectively, with varying thicknesses of evaporated C_{60}, as a function of *q*, which were used to generate **Figure 2g,h** in the main text.

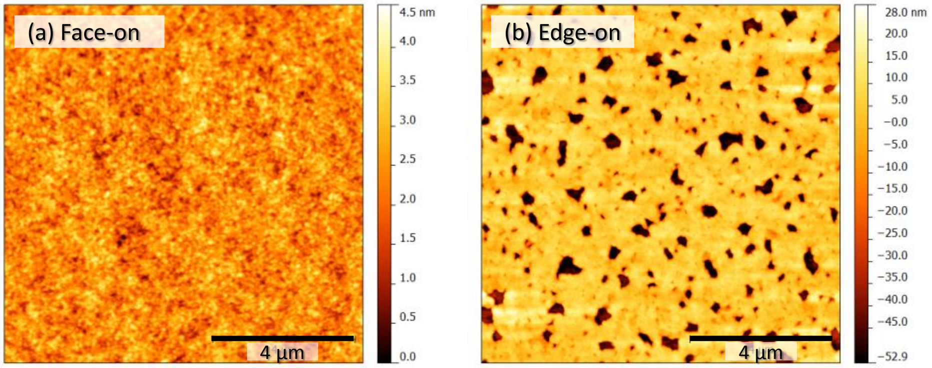

Supplementary Figure 4. AFM topography of p-SIDT(FBTTh$_2$)$_2$. Atomic force microscopy (AFM) images of face-on (a) and edge-on (b) p-SIDT(FBTTh2$_2$)$_2$ films used in the bilayer devices. AFM images were collected in air, using tapping-mode, a Si tip, and an Innova AFM.

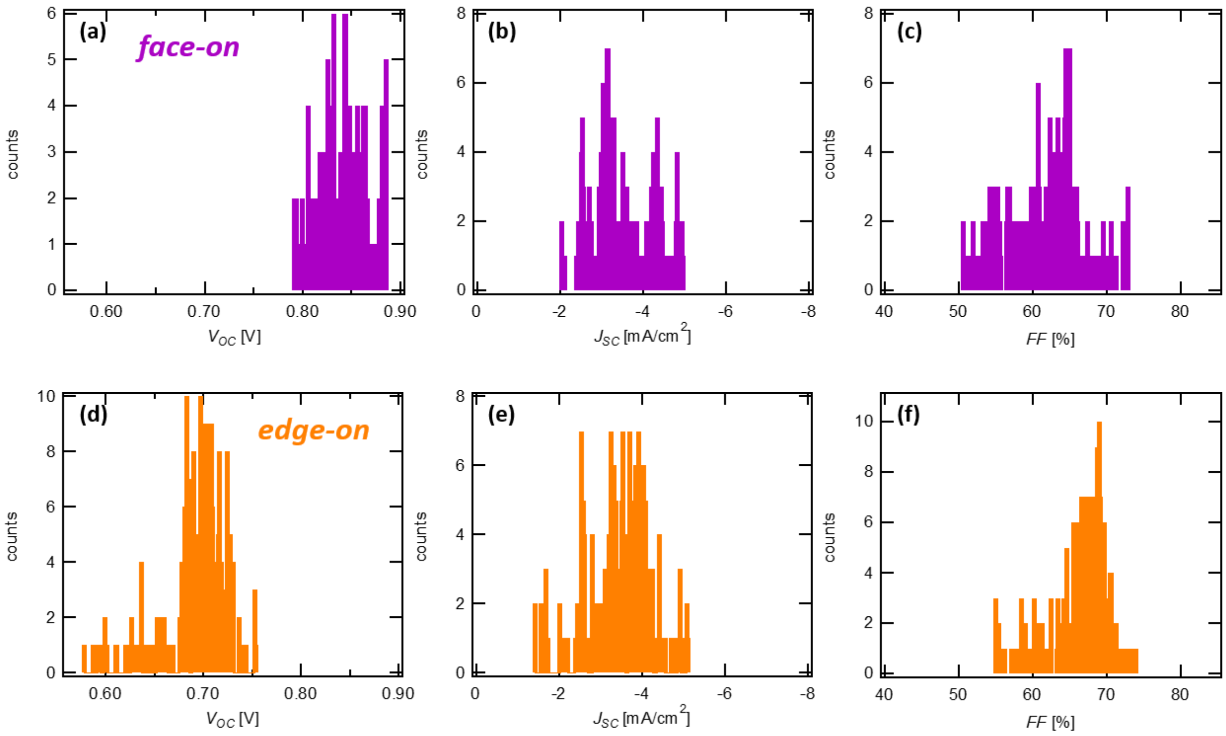

Supplementary Figure 5. Device parameter statistics. Histograms for the V_{OC} (a,d), J_{SC} (b,e), and *FF* (c,f) of face-on (a,b,c,) and edge-on (d,e,f) bilayer devices. Over 150 devices of each orientation were tested. Histograms were generated using automatic binning in Igor.

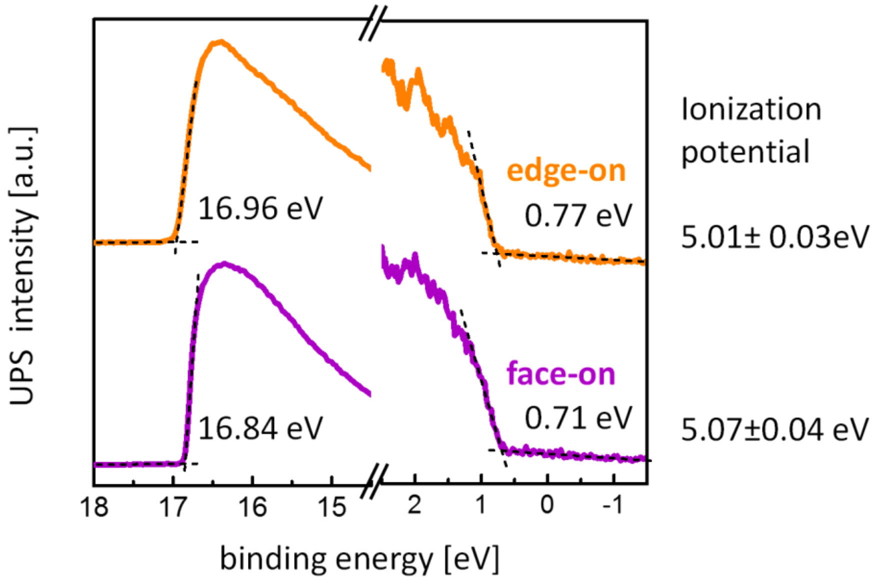

Supplementary Figure 6. Ultraviolet photoelectron spectra of p-SIDT(FBTTh$_2$)$_2$. UPS measurements of neat face-on and edge-on p-SIDT(FBTTh2$_2$)$_2$. UPS measurements were done on very thin films of p-SIDT(FBTTh$_2$)$_2$, approximately 5-8 nm thick, in order to avoid discrepancy in the results due to charging in the semiconductor films. The ionization potential and the respective standard deviation for three samples are reported for each orientation on the right. UPS spectra were measured with a HeI source (21.2 eV), at pressures of low 10^{-7} Pa, a constant pass of 5 eV, current of 10 mA, and the anode set to 6 kV. The ionization potential was calculated according to $I_p = 21.2$ eV – (E_{cutoff} - E_g),[53] where the E_{cutoff} and E_g were determined by the onset at high and low binding energies, respectively. The ionization potential on the right represents the average of 3 repeated measurements and the respective error.

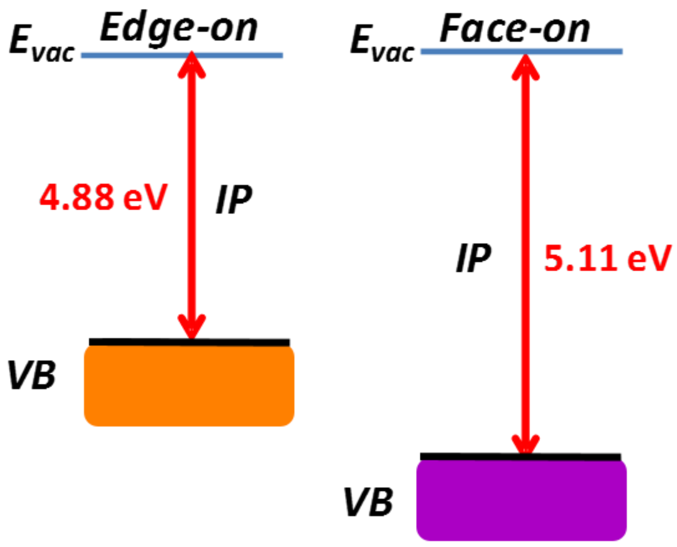

Supplementary Figure 7. Calculated ionization potential of p-SIDT(FBTTh$_2$)$_2$. Ionization potential (IP), evaluated from the vacuum level to the top valence bands (VB) of edge-on (left) and face-on (right) p-SIDT(FBTTh$_2$)$_2$ slabs, estimated at the DFT/HSE level.

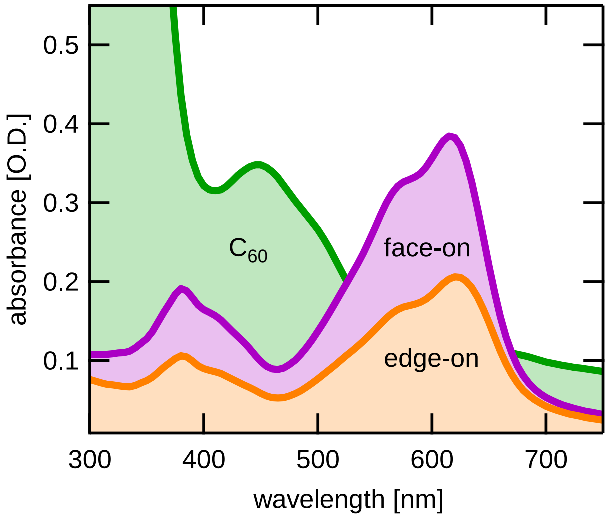

Supplementary Figure 8. Absorption spectra of neat p-SIDT(FBTTh$_2$)$_2$ films. Absorption spectra of neat C_{60}, face-on and edge-on p-SIDT(FBTTh2$_2$)$_2$ films. All films are 45 nm thick, equivalent to the thicknesses used in the bilayer devices tested here.

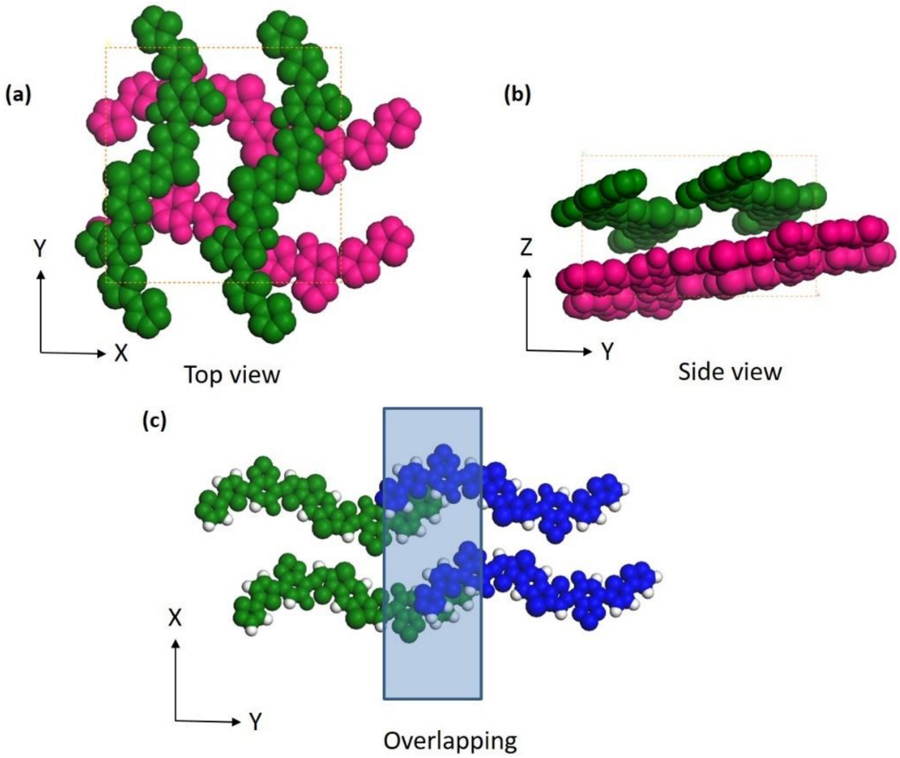

Supplementary Figure 9. Calculated 4-molecule crystal structure of p-SIDT(FBTTh$_2$)$_2$. Crystal structure calculated for p-SIDT(FBTTh$_2$)$_2$, used for electronic structure calculations. (a) Top view of crystal unit cell, (b) side view of crystal unit cell, and (c) an example of the overlap between molecules in adjacent unit cells. Crystal structure was calculated using a combination of molecular dynamics (MD) and Monte Carlo (MC) simulations. The MD simulations were run for 200 ps at 300 K with under the NVT ensemble using the Verlet integrator with a time step of 1 fs. The temperature was maintained by the Nose-Hoover thermostat. A spherical cutoff of 1.25 nm for the summation of van der Waals interactions and the Ewald solver for long-range Coulomb interactions was used throughout. The COMPASS force field as implemented in the Forcite program of Materials Studio was used for the MD simulations.[56] Density-functional theory calculations using the range-separated HSE functional were then carried out for face-on and edge-on p-SIDT(FBTTh$_2$)$_2$ slabs under periodic boundary conditions using the plane-wave based Vienna Ab-initio Simulation Package (VASP). [57-60]

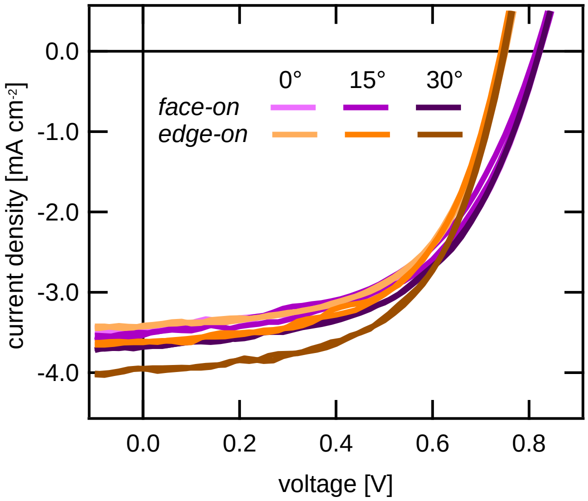

Supplementary Figure 10. *J*-*V* curves as a function of illumination angle. *J*-*V* curves under 1-sun illumination, at illumination angles of 90° (normal to the substrate), 75°, and 60° to the substrate. Illumination angle was varied by rotation the illumination source to the respective angles.

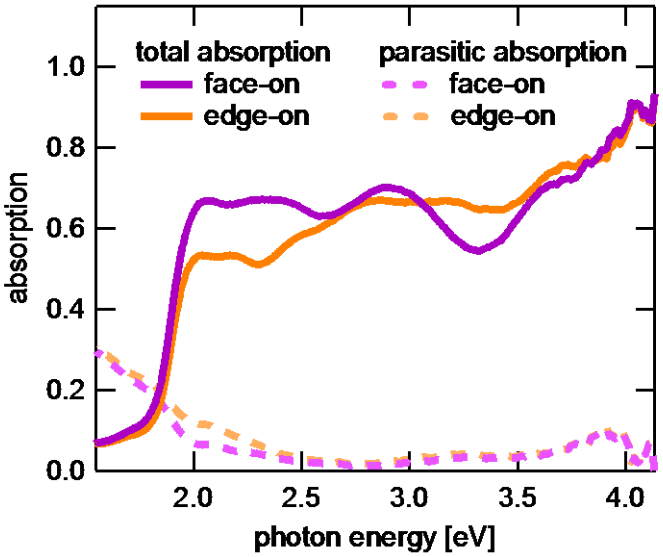

Supplementary Figure 11. Total absorption of bilayer devices. Total absorption, as measured with an integrating sphere, corrected for parasitic absorption from contacts, optical interference and scattering effects, calculated using transfer matrix modeling. We used the Matlab program written by Burkhard *et al.* For more detail, see reference 44 in the main text.

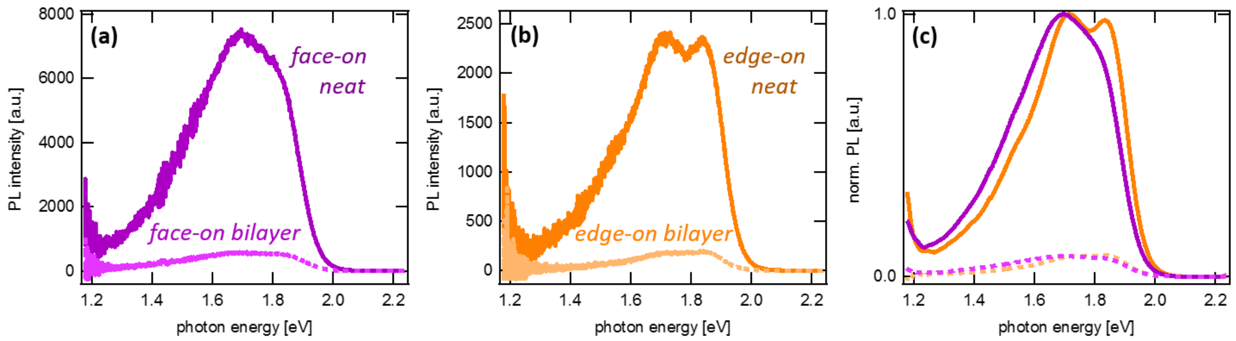

Supplementary Figure 12. PL quenching efficiency as a function of orientation. PL quenching measurements as a function of molecular orientation for neat p-SIDT(FBTTh$_2$)$_2$ and for the bilayer configuration with C_{60} on top for (a) face-on and (b) edge-on films. (c) Smoothed PL spectra, with each orientation normalized to maximum intensity of the neat p-SIDT(FBTTh$_2$)$_2$ film PL to show the relative PL quenching efficiency in each case.

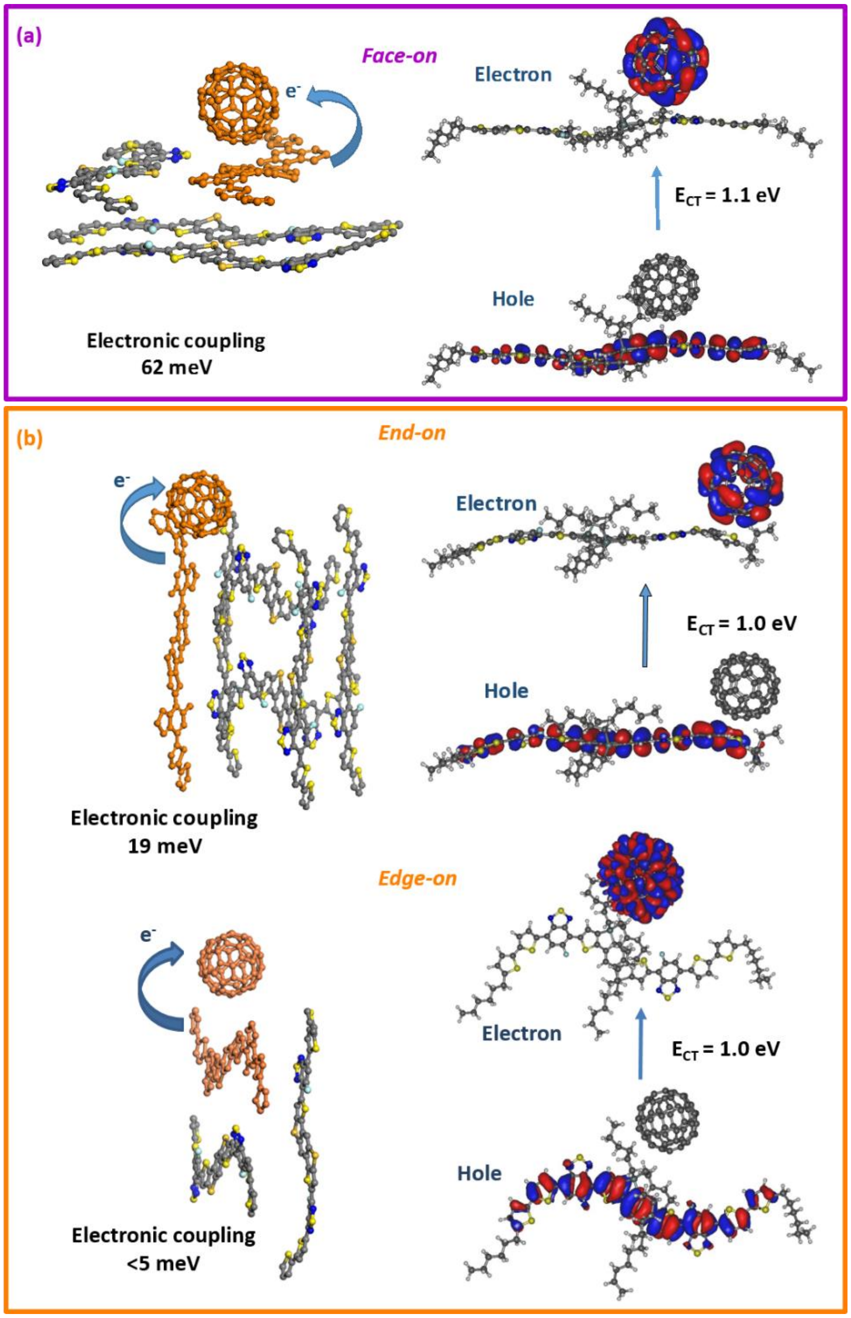

Supplementary Figure 13. Electronic coupling between the lowest charge transfer state and the ground state in p-SIDT(FBTTh$_2$)$_2$/C$_{60}$. Illustration of the face-on (a) or edge-on/end-on (b) p-SIDT(FBTTh$_2$)$_2$/C$_{60}$ configurations that exhibit the largest electronic couplings between the lowest charge transfer state and the ground state. In the case of the end-on molecular orientations, the coupling is as large as 19 meV due to some spatial overlap between C$_{60}$ and the terminal rings on p-SIDT(FBTTh$_2$)$_2$. In all other sampled configurations, including interactions between the side of p-SIDT(FBTTh$_2$)$_2$ and C$_{60}$ (as opposed to the end, as shown here), the calculated electronic coupling was very low. An example of an "edge-on" configuration of p-SIDT(FBTTh$_2$)$_2$/C$_{60}$ is shown at the bottom of panel (b); the electronic coupling in this configuration was found to be less than 5 meV. Left: Donor and acceptor molecules used in the calculations. Right: Natural transition orbitals describing the charge transfer states.

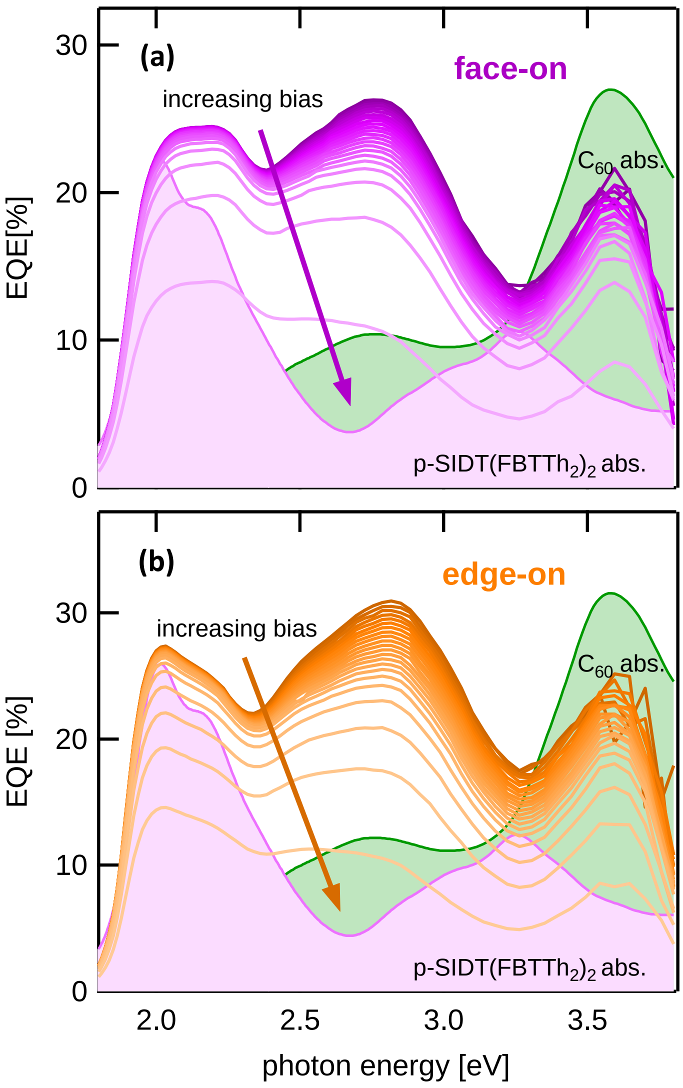

Supplementary Figure 14. Bias-dependent EQE. EQE spectra of (a) face-on and (b) edge-on devices collected under applied bias ranging from V_{OC} to -1.5 V. In the background are unitless absorption spectra of p-SIDT(FBTTh$_2$)$_2$ and C_{60} for reference.

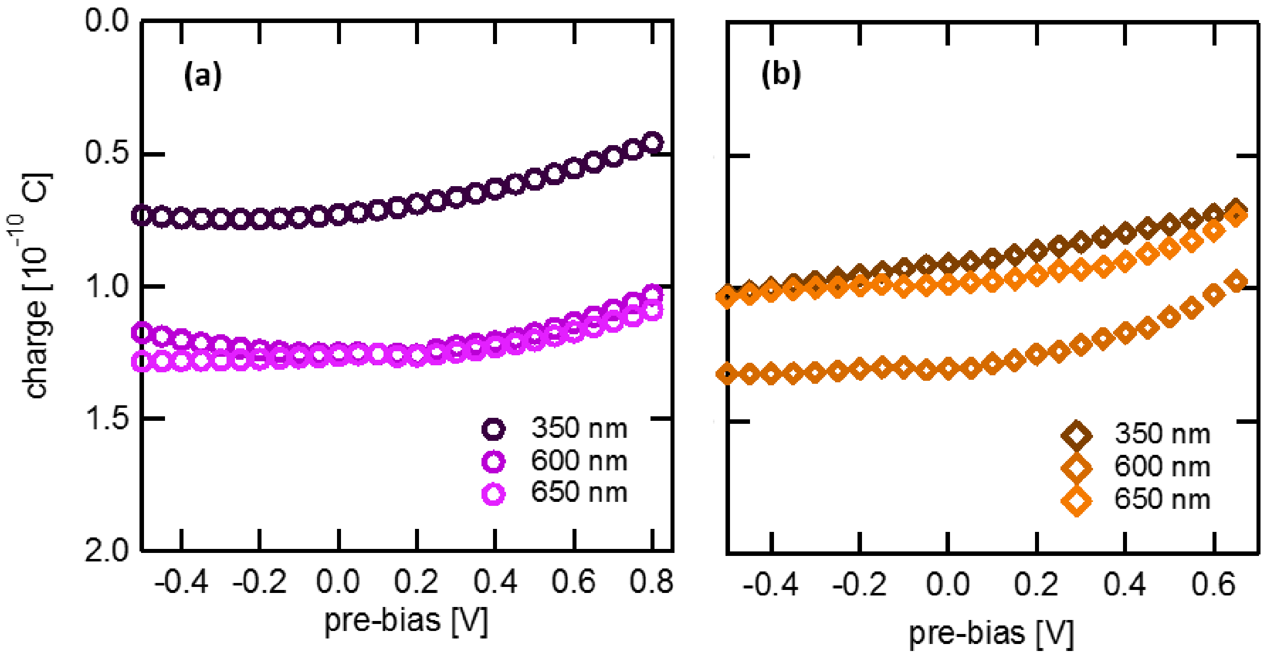

Supplementary Figure 15. Time-Delayed Collection Field (TDCF) measurements. TDCF measurements of (a) face-on and (b) edge-on bilayers excited at varying wavelengths.

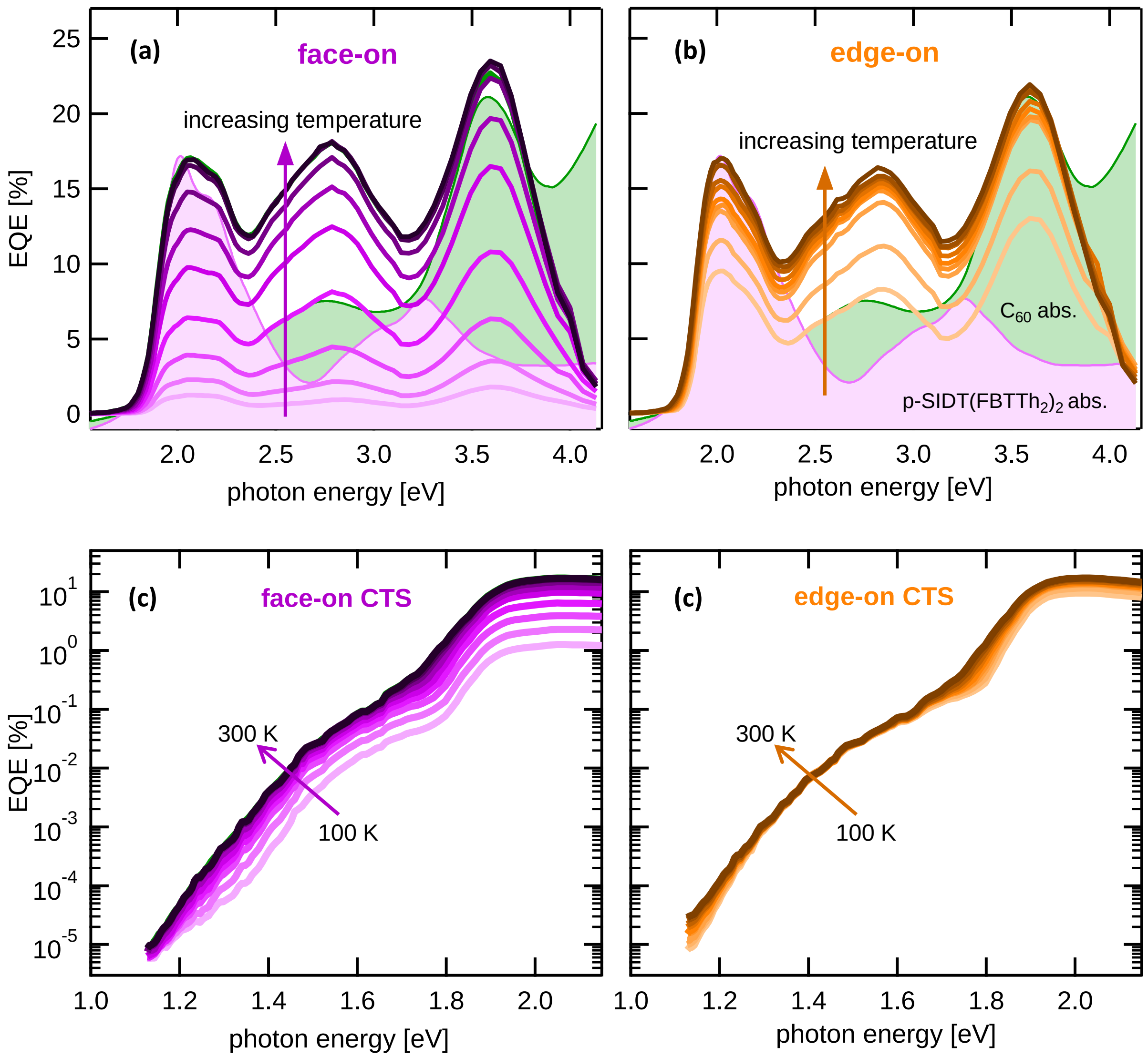

Supplementary Figure 16. Temperature-dependent EQE. EQE spectra collected at varying temperatures ranging from 300 K to 100 K. In the background of (a,b) are unitless absorption spectra of p-SIDT(FBTTh$_2$)$_2$ and C_{60} for reference. (a,b) are the linear EQE spectra over the whole absorption spectrum for face-on and edge-on bilayers, respectively. (c,d) are the EQE spectra on a log-lin scale, at energies corresponding to CTS absorption for the face-on and edge-on bilayers, respectively.

Supplementary Notes

Supplementary Note 1. On the greater order of the edge-on devices.

Devices made on top of annealed face-on films resulted in *s*-shaped curves, analogous to the effect of high contact resistance or the use of non-Ohmic contacts. For this reason, this study is focused on bilayers made from as-cast face-on p-SIDT(FBTTh$_2$)$_2$ compared to edge-on p-SIDT(FBTTh$_2$)$_2$ cast from solutions with DIO (as described in the Methods section). GIWAXS spectra in **Supplementary Figure 2** of face-on p-SIDT(FBTTh$_2$)$_2$ films (b) as-cast and (c) annealed at 100°C for 10 minutes show that annealing the film increases the crystallinity, while maintaining a fully face-on orientation. It is important to note, however, that the GIWAXS and TEM results (in **Figure 2** of the main text) give no indication of amorphous content in the as-cast face-on film. The GIWAXS peaks of the as-cast film are weaker because this film has more disorder over longer length scales, while thermal annealing reduces that disorder and results in more diffraction spots in **Supplementary Figure 2c**, especially at low Q values.

Interestingly, when the full bilayer devices (ITO/PEDOT/p-SIDT(FBTTh$_2$)$_2$/C$_{60}$/BCP/Al) are annealed at 100°C for 10 minutes (**Supplementary Figure 2d,e**), the face-on bilayers do not show *s*-shaped curves until a higher annealing temperature (starting at 116°C). Instead, annealing the full bilayer devices appears to increase the V_{OC} of both the face-on (**Supplementary Figure 2d**) and edge-on (**Supplementary Figure 2e**) devices, while only marginally increasing the J_{SC}. The constant J_{SC} indicates that the quality of the interface is not compromised at these annealing temperatures, and thus the increase in V_{OC} may be a result of altering the contact between the electrodes and semiconductor films. Upon annealing at higher temperatures (above 116°C for face-on devices, and above 120°C for edge-on devices) the J_{SC} begins to increase, and the V_{OC} to decrease, and the face-on devices begin to show an *s*-shaped *J-V* curve. Overall, these data indicate that the results to follow are not influenced by the difference in the crystallinity or long-range order of the face-on and edge-on devices: the V_{OC} is consistently higher for the face-on device, and the J_{SC} consistently similar between the two.

Supplementary Note 2. On the role of pinholes in changing device performance:

Addressing the role of the pinholes (seen in the AFM, Supplementary Figure 4b) on the V_{OC} in the edge-on devices, we consider two ways that the pin-holes could affect the V_{OC}. First, it may be that an interaction between the C$_{60}$ and PEDOT may cause a V_{OC} between the LUMO of C$_{60}$ and PEDOT, which may alter the V_{OC} of the bulk device. Therefore, if one were to change the work function (WF) of the bottom contact (PEDOT), the V_{OC} of this device should also change. We have made the same bilayer devices on MoOx (which has a deeper WF than PEDOT), but the device characteristics did not change, indicating that this is not a dominant effect. Second, the pinholes expose vertical surfaces of the underlying film where the molecules may be face-on with respect to the donor/acceptor interface. In this scenario, one may consider that the V_{OC} of the edge-on film will be higher than a device with a purely edge-on donor/acceptor interface. An estimation of the possible contribution from these face-on interfaces to the V_{OC} can be obtained by estimating the V_{OC} in the devices as a linear combination of the available interfaces. If the area of the device is 0.15 cm^2, and 10% of this area is missing due to pinholes (as is estimated from the AFM scans of the edge-on films, **Supplementary Figure 4b**), then the actual area of the edge-on interface is (0.15-0.015)=0.135 cm^2. However, now there is contact between C$_{60}$ and the walls of the pinholes, where p-SIDT(FBTTh$_2$)$_2$ will be face-on at the interface with C$_{60}$. Assuming there are 100 pinholes per device, and the donor layer thickness is 45 nm (true for the devices in this manuscript), the face-on surface area of due to the pinholes comes out to be 6.2E-6 cm^2. The donor/acceptor surface area that is face-on in these devices

is therefore 0.005% of the total donor/acceptor surface area, and therefore we consider it to have a negligible effect on our results. Therefore, we do not believe the pinholes play a significant role in altering the results and conclusions of this study.

Supplementary References

1. Rivnay, J., Mannsfeld, S. C. B., Miller, C. E., Salleo, A. & Toney, M. F. Quantitative Determination of Organic Semiconductor Microstructure from the Molecular to Device Scale. *Chem. Rev.* **112,** 5488–5519 (2012).
2. Seo, J. H. & Nguyen, T.-Q. Electronic Properties of Conjugated Polyelectrolyte Thin Films. *J. Am. Chem. Soc.* **130,** 10042–10043 (2008).
